# Innate and Conditioned Responses to Chemosensory and Visual Cues in Asian Citrus Psyllid, *Diaphorina citri* (Hemiptera: Liviidae), Vector of Huanglongbing Pathogens

**DOI:** 10.3390/insects5040921

**Published:** 2014-11-19

**Authors:** Joseph M. Patt, Dara Stockton, William G. Meikle, Mamoudou Sétamou, Agenor Mafra-Neto, John J. Adamczyk

**Affiliations:** 1 USDA-Agricultural Research Service, U.S. Horticulture Research Laboratory, 2001 South Rock Road, Fort Pierce, FL 34945, USA; 2 Lake Alfred Research and Education Center, University of Florida, 700 Experiment Station Road, Lake Alfred, FL 33850, USA; E-Mail: dara.stockton@ufl.edu; 3 USDA-Agricultural Research Service, Carl Hayden Bee Research Center, 2000 East Allen Road, Tucson, AZ 85719, USA; E-Mail: william.meikle@ars.usda.gov; 4 Citrus Center, Texas A&M University at Kingsville, 312 N. International Blvd., Weslaco, TX 78596, USA; E-Mail: mamoudou.setamou@tamuk.edu; 5 ISCA Technologies, Inc., 1230 W. Spring Street, Riverside, CA 92507, USA; E-Mail: president@iscatech.com; 6 USDA-Agricultural Research Service, Southern Horticultural Laboratory, P.O. BOX 287, Poplarville, MS 39470, USA; E-Mail: john.adamczyk@ars.usda.gov

**Keywords:** associative learning, plant-herbivore interactions, host-plant selection behavior, olfaction, bioassay, citrus, IPM, citrus greening disease

## Abstract

Asian citrus psyllid (*Diaphorina citri*) transmits Huanglongbing, a devastating disease that threatens citrus trees worldwide. A better understanding of the psyllid’s host-plant selection process may lead to the development of more efficient means of monitoring it and predicting its movements. Since behavioral adaptations, such as associative learning, may facilitate recognition of suitable host-plants, we examined whether adult *D. citri* could be conditioned to visual and chemosensory stimuli from host and non-host-plant sources. Response was measured as the frequency of salivary sheaths, the residue of psyllid probing activity, in a line of emulsified wax on the surface of a test arena. The psyllids displayed both appetitive and aversive conditioning to two different chemosensory stimuli. They could also be conditioned to recognize a blue-colored probing substrate and their response to neutral visual cues was enhanced by chemosensory stimuli. Conditioned psyllids were sensitive to the proportion of chemosensory components present in binary mixtures. Naïve psyllids displayed strong to moderate innate biases to several of the test compounds. While innate responses are probably the psyllid’s primary behavioral mechanism for selecting host-plants, conditioning may enhance its ability to select host-plants during seasonal transitions and dispersal.

## 1. Introduction

The Asian citrus psyllid, *Diaphorina citri* (Kuwayama) (Hemiptera: Liviidae), transmits *Candidatus* Liberibacter asiaticus (*C*Las), the causal agent of citrus greening disease or Huanglongbing [1,2]. This devastating disease threatens citrus production worldwide and has resulted in the loss of hundreds of thousands of hectares of orchards [3,4]. *Diaphorina citri* ingests primarily phloem sap; it mates, reproduces and develops only on the growing tips of terminal shoots of *Citrus* and closely related genera, such as *Murraya* and *Bergera* [1,2,3]. *Diaphorina citri* can move over large distances transmitting *C*Las from one area to another in a short amount of time [5,6,7,8].

Detection and monitoring programs for *D. citri* are a critical component of area-wide management programs aimed at suppressing psyllid populations and slowing the spread of *C*Las [1,2]. Along with tapping samples and visual inspections, current detection and monitoring efforts for *D. citri* rely heavily upon sticky card traps [9,10]. The efficacy of these traps is questionable, however, especially when population densities of the psyllid are low [11]. There is a strong need to improve our ability to track *D. citri* to accurately locate new infestations, monitor its range expansion, and evaluate suppression tactics. One possible means of improving trap efficacy is the development of potent scent lures based on host-plant attractants. At present, field tests of synthetic scent baits have produced ambiguous results; scent mixtures tested so far have been inconsistent in attracting *D. citri* to sticky card traps [12]. The formulation of potent scent lures has been hampered by a lack of understanding of the host-plant selection behavior of *D. citri*.

Visual cues play a prominent role in host-plant location by *D*.* citri*; it is especially attracted to wavelengths corresponding to bright yellow and green in the human visible spectrum [13] and sticky card traps used to monitor the psyllid are bright yellow or yellowish green in color [9,10,11,12,13]. Wenninger *et al.* [14] found that *D. citri* responded to visual stimuli in the absence of host-plant odor, suggesting that visual cues alone are sufficient to elicit attraction to host-plants. Nonetheless, *D. citri* is stimulated by host-plant odors [14,15,16,17,18] and olfaction seems to play some role in host-plant selection. The nature of that role, however, is not clear; *i.e.*, while host-plant odor itself may not function as an orientation cue, it may enhance psyllid responsiveness to visual cues. For example, in a laboratory study, psyllid response to gray-colored visual targets was enhanced by the addition of scent cues but this effect was not observed with yellow-colored targets [16]. Response to host-plant odors is concentration dependent and *D. citri* can distinguish between mixtures comprised of three different monoterpenes [16]. Further evidence that olfactory cues can play a significant role in host-plant selection behavior is provided by the discovery that *C*Las can alter foliar odor, making infected hosts more attractive to psyllids than uninfected plants [19]. This effect has been observed in other psyllid-pathogen-host-plant associations as well [20,21,22].

*Diaphorina citri* is dependent on emerging shoots for its reproduction; efficient discrimination between actively growing and non-growing host-plants may require the acquisition, integration, and retention of information conveyed by a number of different stimuli [15,16]. The foliar odors of *Citrus* and related genera are comprised primarily of mono- and sesquiterpenes but their compositions vary greatly among species [15,23,24]. In addition to interspecific differences , the volatile components of foliar odors can vary qualitatively and quantitatively due to physiological condition [25,26,27] and growth stage [28,29,30,31,32].

Behavioral adaptations, such as associative learning, may facilitate recognition of developmentally suitable host-plants within the psyllid’s general environment. For example, if the volatile profile of a host-plant predictably indicates the presence of growing shoots, then that particular volatile profile may function as a conditional stimulus to *D. citri*. In other words, *D. citri* could learn to associate a particular volatile profile with a particular host’s growth stage while feeding or ovipositing. A particular volatile profile could then function as a cue to help the psyllid discriminate between developmentally-suitable and unsuitable foliage while searching for mates or oviposition sites. This recognition of foliar volatiles could potentially occur during the immature stage [33,34,35]. Prager *et al.* [35] showed that the selection of host-plants for oviposition made by female potato psyllids, *Bactericera cockerelli* Sŭlc, is based, in large part, by the female’s natal host-plant; this also applies to the settling behavior of both sexes. The cognitive abilities of *D. citri* might be an important consideration in the development and deployment of effective scent lures to improve detection and monitoring. For example, if a psyllid forages on a particular host-plant species, then its experience with the chemosensory stimuli associated with that host-plant may predispose it to be more attracted to a scent lure that mimics that particular host-plant. Alternatively, if innate attraction to chemosensory stimuli strongly biases psyllid response, then a generalized scent lure comprised of host-plant compounds known to elicit strong innate responses may be more effective.

The goal of the work reported here was to determine whether adult *D. citri* could associatively learn to recognize chemosensory stimuli, both from host- and non-host-plant sources. A secondary purpose was to determine whether conditioning tests could be used to reveal behavioral responses to chemosensory compounds that are not expressed by tests of innate response. The foliar odors of many *D. citri* host-plants are comprised primarily of mono- and sesquiterpenes [23,24]. Limonene, a monoterpene hydrocarbon present in the foliage of numerous *Citrus* species [23,24], was selected as a representative semiochemical of *D. citri* host-plants. Menthol, a monoterpene alcohol produced in the foliage of many mint species [36] but which is absent from *Citrus* leaves [24], was selected as a representative non-host-plant monoterpene. Isoamyl acetate, a common component of fruit aromas and flavors, was selected as a representative fruit ester. Of these three compounds, only limonene, because of its prevalence in *Citrus* foliage, was expected to elicit a strong innate response from *D. citri*.

**Figure 1:**
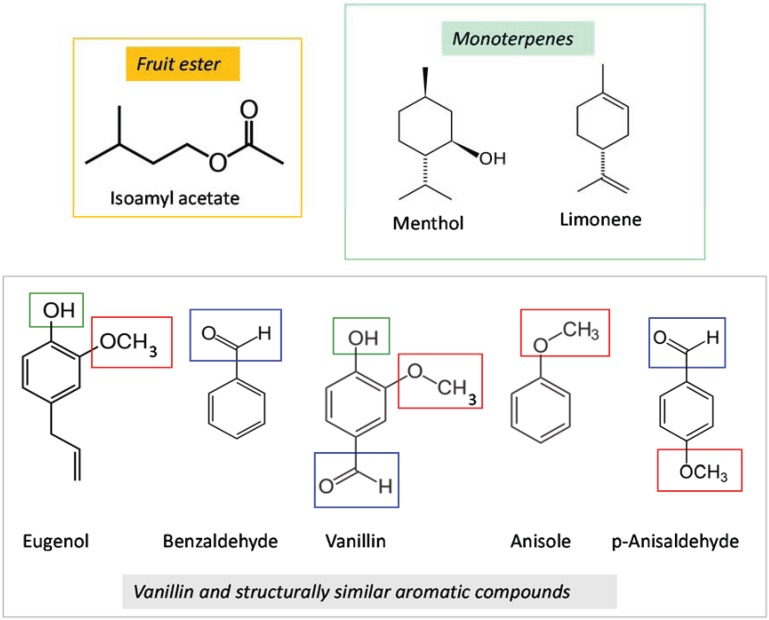
Chemical classes of test compounds.

With the exception of methyl anthranilate, aromatic compounds (*sensu* arenes) are largely absent from *Citrus* foliage [24]. Vanillin, a phenolic aldehyde, was used in the initial learning test as the conditioned stimulus because *D. citri* was expected to be naïve to vanillin and thus have a neutral response to it. Neither vanillin nor any of the other aromatic test compounds listed below were detected in the foliar aromas of the colony plants [15]. Because of the psyllid’s strong conditioned response to vanillin, subsequent tests examined *D. citri* response to a selected group of aromatic compounds having the same functional groups, and thus a similar molecular geometry, as vanillin: benzaldehyde (1C carbonyl), anisole (1C methoxy), p-anisaldehyde (1C carbonyl + 4 C alcohol), and eugenol (l C alcohol + 2C methoxy) (Figure 1). These compounds are constituents of the foliage and fruits of many non-host plant taxa and elicit behavioral responses in particular insect herbivores and pollinators. As with vanillin, their rarity in *Citrus* foliage made them good candidates for testing associative learning in *D. citri* because the psyllids were expected to be naïve towards them. We also examined whether mixing and concentration of conditional stimuli had an effect on conditioned response. Lastly, conditioned response to a neutral color and neutral color + scent combination was evaluated.

## 2. Materials and Methods

### 2.1. Experiment Overview

During feeding, *D. citri* secrete a salivary sheath that facilitates passage of the mouthparts into the vascular bundles [37]. Because the salivary sheath can be stained and visualized with Coomassie blue [16,38], it was possible to measure a psyllid’s probing frequency into an artificial wax substrate containing an olfactory or visual test stimulus. The response variable used in all experiments was the frequency of probing events, measured as the number of salivary sheaths observed in a line of emulsified wax substrate drawn across the surface of a test arena [16]. In developing this assay, we used the six-stage sequence of host-plant selection behaviors described for aphids [39] as a guide.

A dose-response experiment was conducted on each test stimulus to evaluate the innate probing response of naïve psyllids to the test stimulus and to determine if probing response was affected by test stimulus concentration (Table 1). This was used to identify whether the test compounds were innately stimulatory, inhibitory or neutral relative to a blank control. Based on those results, subsequent experiments were then conducted to determine whether experience with the test compounds could modify psyllid response. Classical conditioning is traditionally defined as the pairing of an unconditioned stimulus (US) with a neutral conditioned stimulus (CS) to produce a conditioned response (CR) (Table 1). The experimental protocol for conditioning consisted of two stages: (1) A conditioning stage, in which the psyllids were fed either plain sucrose solution (= unconditioned stimulus (US)) or a sucrose solution + a test CS (= US + conditioned stimulus (CS)); and, (2) A testing stage, which compared probing frequencies between naïve and experienced *D. citri* on blank wax lines or wax lines + the test CS. In a follow up study, a test was conducted to determine whether *D. citri* could be conditioned to associate a neutral color (blue) when paired either with sucrose solution alone or a sucrose solution + a general citrus scent.

**Table 1:** Overview of experiments performed.

Behavior test	Stimuli tested
**Innate response tests (Dose-response)**	*Monoterpenes:* Limonene, Menthol
	*Fruit Ester:* Isoamyl acetate
	*Aromatics:* Vanillin, Anisole, Anisaldehyde, Eugenol, Benzaldehyde
**Conditioned response to individual compounds**	
**Trained on: US blank or US + CS**	*US:*0.3 M Sucrose solution
**Tested on: US blank or US + CS **	*CS:* Vanillin, Limonene, Isoamyl acetate, Eugenol
**Conditioned response to concentration**	*US:* 0.3 M Sucrose solution
	*CS:* Benzaldehyde (3 µL or 10 µL/10 mL SPLAT)
**Conditioned response to binary mixtures**	*US:* 0.3 M Sucrose solution
	*CS:* 1:1 Vanillin: Isoamyl acetate, 1:2 Vanillin: Isoamyl acetate, 2:1 Vanillin: Isoamyl acetate
**Conditioned response to blue-colored feeding response with and without generic citrus chemosensory cue**	*US:* 0.3 M Sucrose solution
	*CS:* Blue food coloring ± Generic citrus scent

### 2.2. Test Compounds

Imitation food flavorings were used as source material for vanillin (vanilla extract (Adams Extract Co. Inc., Austin, TX, USA)), isoamyl acetate (banana extract), and benzaldehyde (almond extract) (both McCormick & Co., Inc., Hunt Valley, MD, USA) (Table 2). Analysis with gas chromatography-mass spectrometry showed that the flavorings contained the CS of interest as the sole active ingredient, with aqueous ethanol as the carrier solvent. Since menthol is a solid at room temperature, a stock solution was made by dissolving 55 mg menthol in 80 mL ethanol. The remaining test compounds were all reagent grade liquids (SAFC, St. Louis, MO, USA) and added directly into the emulsified SPLAT on a volume to volume basis. Using the results of a previous study as a guide [16], the concentrations of the stock solutions ranged from 0.3 µL to 1.0 µL test compound per 1 mL sucrose solution (Table 2). Since we have shown that *D. citri* is sensitive to concentration effects [16], lower stock solution concentrations of some compounds were used when it was deemed that higher concentration levels (*i.e.*, 1.0 µL/1 mL) might be repellent.

**Table 2:** Compounds used in associative learning tests, their concentrations used during the conditioning and testing phase of the tests, and their commercial sources.

Test Compound	Conditioning Phase Concentration (µL/100 mL Sucrose Solution)	Probing Test Concentration (µL/10 mL Emulsified Wax)
Vanillin	100	40
Isoamyl acetate	100	20
Benzaldehyde	100	3 and 30
Limonene	100	20
Eugenol	20	20
Anisaldehyde	3	20
Anisole	20	20

### 2.3. Study Insects

*Diaphorina citri* were reared at the former USDA-ARS laboratory in Weslaco, TX. Colony host-plants included Mexican lime (*C*. *aurantifolia* (Christo.) Swingle), Meyer lemon (*Citrus × limon* L. Burm. f.), and orange jasmine (*Murraya paniculata* (L.) Jack.). Psyllid used in the tests were 7–21 days post emergence; post-hoc examination of the psyllids showed that males and females were equally represented in the tests. The psyllids were collected on the morning of the test and held for 2 h in a lighted incubator at 25 ± 2 °C.

### 2.4. Innate Response Tests

**Figure 2:**
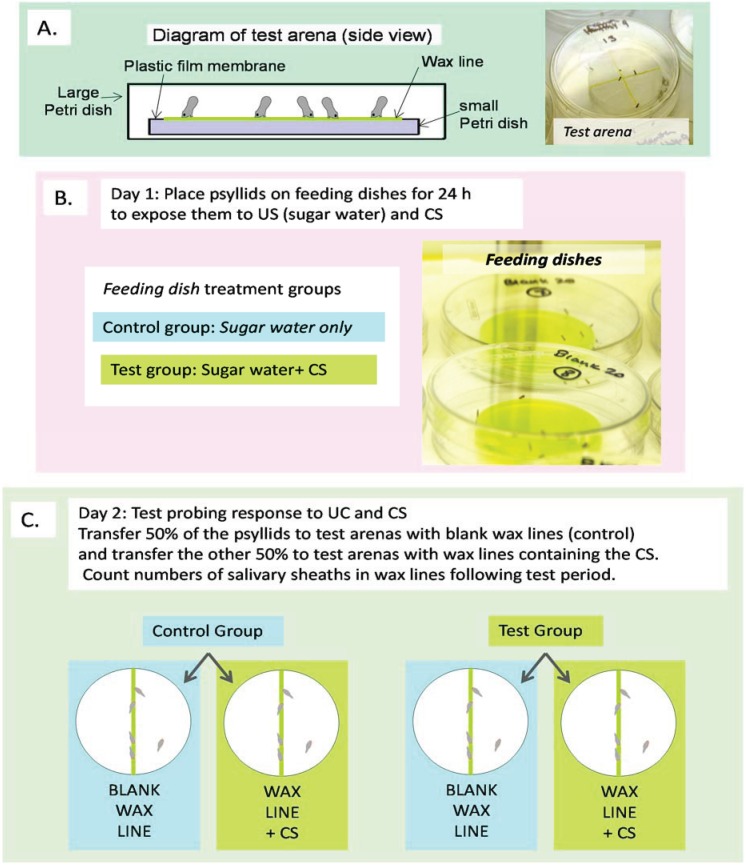
Schematic of experimental procedure. (**A**) Cross section view of test arena used in both innate and conditioned response tests. Photo insert shows psyllids on SPLAT lines. (**B**) Conditioning protocol. (**C**) Conditioned response test protocol.

A test arena that mimicked a leaf surface [16] was used to measure the psyllids’ innate probing response to the CS. The test arena consisted of a 4 × 4 cm piece of Parafilm (American National Can, Inc., Chicago, IL, USA) stretched over the opening of a 52 mm dia plastic Petri dish (Becton Dickenson Labware, Franklin Lakes, NJ, USA). Using a syringe and a 20G needle, a thin line of an emulsified wax (SPLAT^™^, ISCA Technologies, Inc., Riverside, CA, USA) was drawn across the membrane (Figure 2A). SPLAT is used to dispense pheromones and other semiochemicals in crops [40,41]; in this study, test CS were mixed into the SPLAT prior to application. To facilitate visual location by the psyllids, the SPLAT lines were tinted pale green with “neon green” food coloring (McCormick & Co., Inc.) (6 µL food coloring/10 mL SPLAT) [16]. Psyllids were exposed to test CS at four different concentrations based on a half-log scale: 3 µL-, 9 µL-, 30 µL-, and 90 µL aliquots of test CS were mixed into 10 mL SPLAT. The half-log scale was selected because preliminary experiments indicated a strong repellent effects at higher log-based concentrations and we have shown previously that *D. citri* can discriminate aromatic compounds within this range of concentrations [16]. Psyllids in the control treatment were exposed to blank SPLAT. The control treatment in the menthol test contained 90 µL ethanol/10 mL SPLAT to compensate for the presence of ethanol in the menthol stock solution.

To facilitate transfer and prevent trauma, the psyllids were anaesthetized by chilling immediately prior to the start of each trial. Five individuals were placed in each test arena; they were confined by placing the test arena inside a larger plastic Petri dish (9 cm dia × 1.5 cm tall) (Becton Dickenson Labware). The dishes were placed in an incubator set at a temperature of 28 °C (± 1 °C) for 2 h. To maintain consistency, all experiments were conducted between 11:00 and 13:00. At the end of each trial, the psyllids were euthanized by chilling. Post-mortem examinations were conducted to determine the number of males and females in each dish. The dishes were immersed in Coomassie blue (Sigma-Aldrich Chemical Co., St. Louis, MO, USA) solution (0.1% Coomassie blue R250, 20% (v/v) methanol, and 10% (v/v) acetic acid) for 2 minutes, then rinsed twice with reverse osmosis water, and air-dried. Probes were quantified manually with a stereomicroscope at 4× magnification level. Thirty replicates were performed for each concentration of vanillin, benzaldehyde, isoamyl acetate, menthol, and anisaldehyde, 15 replicates for each concentration of limonene and eugenol, and 45 replicates for each concentration of anisole.

### 2.5. Learning Test

#### 2.5.1. Learning Test Part 1: Conditioning Procedure

The conditioning experiment had four treatment groups which encompassed the different possible US + CS combinations plus the necessary blank controls (Figure 2B,C):

(1) Fed blank sucrose solution (unconditioned stimulus (US)), then tested on blank SPLAT (US → blank); this treatment provided a baseline level of probing by naïve psyllids exposed only to the US.
(2) Fed blank sucrose solution then tested on SPLAT + test CS (US → CS); this treatment measured the level of probing by naïve psyllids exposed to the CS only during the testing phase.
(3) Fed sucrose solution + test CS then tested on blank SPLAT (US + CS → blank); this treatment measured the level of probing by psyllids that had been exposed to both US + CS during the conditioning phase.
(4) Fed sucrose solution + test CS then tested on SPLAT + CS (US + CS → CS); this treatment measured the response of psyllids to the CS following exposure to it during the conditioning phase.

The US and test CS were presented to the psyllids via a feeder dish (Figure 2B). The feeder dish consisted of a 4 × 4 cm piece of Parafilm stretched over the opening of a 52 mm dia plastic Petri dish. The psyllids could penetrate the membrane with their stylets and ingest the test solution contained within the dish. Test solutions consisted of 0.3 M aqueous sucrose solution (US) plus an aliquot of the test compound (CS) (Table 1); the control treatment for all tests was blank sucrose solution. To induce probing by the psyllids, the sucrose solution was tinted green with “neon green” food coloring (McCormick & Co., Inc.) (50 µL food coloring/40 mL sucrose solution). Between eight and fifteen psyllids were released onto each feeder dish. The psyllids were confined by placing the feeder dish inside a larger plastic Petri dish (9 cm dia × 1.5 cm tall). The conditioning period lasted 24 h and began between 8:00 and 9:00. The dishes were held in an incubator set at a temperature of 28 °C (± 1 °C) and a 14:10 (L:D) photoperiod.

#### 2.5.2. Learning Test Part 2: Probing Response Procedure

The same type of test arena used to measure innate response was used to measure probing response to the test CS (Figure 2C). The concentrations of the test CS were selected based on the results of the innate response tests (Table 1). Following the 24 h conditioning period, the psyllids were anaesthetized by chilling and five individuals were transferred to a test arena. Forty-five replicates were performed for each treatment and accompanying control for anisaldehyde, 40 replicates for vanillin, amyl acetate, and anisole, and 30 replicates for limonene and eugenol.

### 2.6. Conditioned Response to the Same CS at Two Concentrations

Following conditioning to benzaldehyde (100 µL benzaldehyde/40 mL sucrose solution), psyllid response was measured to SPLAT lines with either a low (3 µL benzaldehyde/10 mL SPLAT) or high concentration (20 µL benzaldehyde/10 mL SPLAT) of benzaldehyde. In this design there was no blank SPLAT treatment. Thirty replicates were performed for each concentration treatment and accompanying control.

### 2.7. Effect of Mixing on Conditioned Response

Tests were conducted to determine if the addition of a second compound interfered with the responses of conditioned psyllids to the CS. In this test, psyllid response to mixtures of vanillin and isoamyl acetate was measured after the psyllids were conditioned to sucrose solution + vanillin. Isoamyl acetate was selected because the innate and conditioned response tests showed that, while the psyllids perceive it, experience did not affect probing frequency. On the other hand, psyllids demonstrated an increase in probing frequency following feeding experience with vanillin. In these tests, all of the psyllids were conditioned to vanillin, as described above. Probing frequency was then measured in no-choice assays with SPLAT lines containing 1:1, 1:3, or 3:1 mixtures of vanillin and isoamyl acetate, respectively. A blank SPLAT treatment was included in the study design as a negative control as were positive controls of vanillin (40 µL flavoring/10 mL SPLAT) and isoamyl acetate (20 µL flavoring/10 mL SPLAT). Thirty replicates were performed for each treatment and the control.

### 2.8. Conditioned Response to Visual Cues

A conditioning dish protocol similar to that used in the semiochemical learning assays was used to determine whether psyllids associatively learn to link blue, a non-stimulatory color, to the presence of food. Blue was chosen as a test color because in field studies, blue colored sticky card traps were not effective in capturing *D. citri* [13]. During the conditioning phase, 6–20 psyllids were confined to feeder dishes containing the following treatments: (1) Aqueous blue solution (50 µL blue food coloring (McCormick) /40 mL RO water), (2) Aqueous blue solution + sucrose (50 µL blue food coloring/12 g sucrose/40 mL RO water), (3) Aqueous blue solution + citrus leaf scent mixture (50 µL blue food coloring/2 µL scent mixture/40 mL RO water), and (4) Aqueous blue solution + citrus leaf scent + sucrose (50 µL blue food coloring/2 µL scent/12 g sucrose/40 mL RO water). The scent was a mixture of common citrus volatiles found to be innately stimulating to psyllids [15]. The psyllids were exposed to the conditioning solution for 24 h in an incubation chamber as in the conditioning tests above. After 24 h, the psyllids were transferred to arenas with a SPLAT line colored blue (50 µL food coloring/5 mL SPLAT). Because the base color of the SPLAT was white, the blue-colored SPLAT lines were about 30% brighter than water-based conditioning solution; however, spectral analysis (Ocean Optics, Dunedin, FL, USA) showed that both the conditioning solution and SPLAT lines had similar spectra, with absorption/reflectance maxima at 450 nm and 700 nm. All SPLAT lines were scentless, isolating color as the only stimulus prompting probing behavior. As a control, one-third of the arenas from all four conditioning treatments were prepared with one line of bright green SPLAT (50 µL neon green coloring/5 mL SPLAT). These dishes were distributed randomly in the incubator across all treatments. Unlike the previous experiments, only one psyllid was transferred per dish due to high overnight mortality. Ten psyllids were tested for each treatment.

### 2.9. Data Analysis

Innate responses were evaluated with one-way ANOVA (α = 0.05) for differences between treatments. If F-values were significant, then *t*-tests of post-hoc orthogonal comparisons were used to look for significant differences between individual pairs of treatments [42]. Likewise, conditioned responses were analyzed by planned comparison *t*-tests for all pair-wise comparisons of test treatments (naïve tested on blank SPLAT; naïve tested on SPLAT + test CS, experienced tested on blank SPLAT, and experienced tested on SPLAT + test CS). For all analyses, the Bonferroni correction (α = 0.05/n treatments) was employed to address the experiment-wise error rate [42]. Because sex was recorded *post hoc*, possible correlations between psyllid sex and probing frequency were evaluated with linear regression (α = 0.05).

## 3. Results

### 3.1. Innate Response

**Figure 3:**
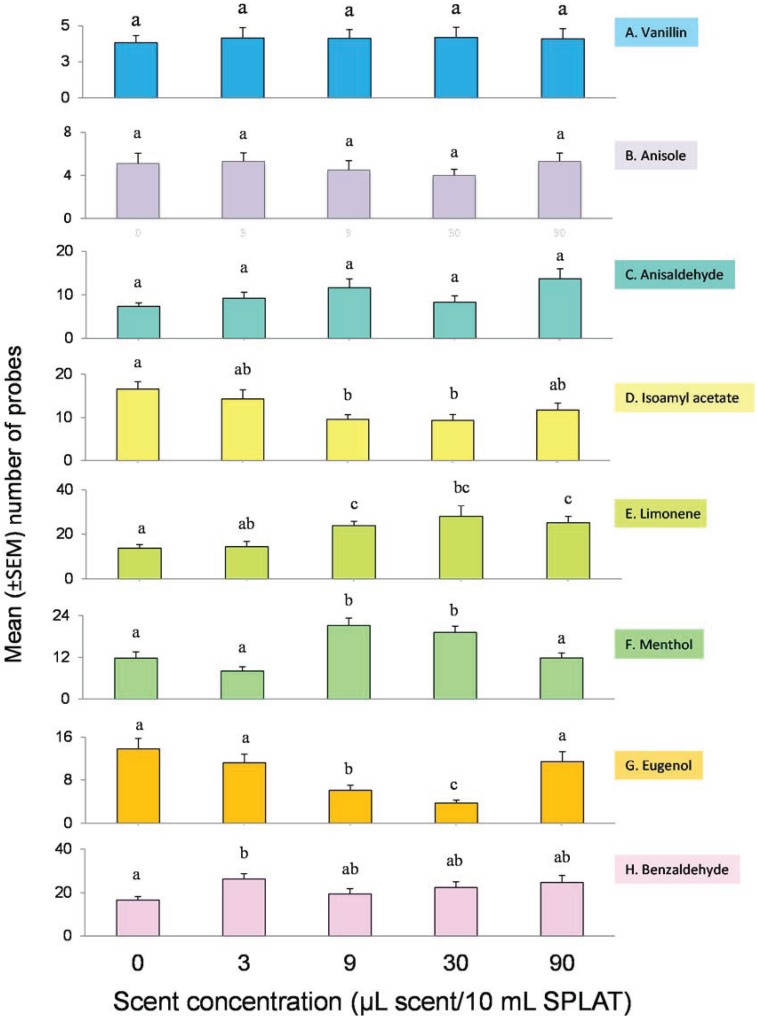
Innate probing responses of psyllids to test compounds at different concentrations. Letters within the same row are different at *p* ≤ 0.05 (Planned comparisons with t-tests). Note differences in y-axis scales. N = 30 for all compounds except for limonene and eugenol (N = 15) and anisol (N = 45). Sample size is replicated for each dose within each olfactory stimulus.

The number of probes made by naïve psyllids into SPLAT lines containing different concentrations of test compounds did not differ significantly across concentrations in the vanillin (*F*_(4,145)_ = 0.05, *p* = 0.995), anisole (*F*_(4,220)_ = 1.73, *p* = 0.145), or anisaldehyde (*F*_(4,145)_ = 2.263, *p* = 0.065) treatments (Figure 3A–C). Significant difference were observed in probing frequency at different concentrations of isoamyl acetate (*F*_(4,143)_ = 3.821, *p* = 0.006), limonene (*F*_(4,70)_ = 4.952, *p* = 0.001), menthol (*F*_(4,145)_ = 10.296, *p* < 0.001), eugenol (*F*_(4,70)_ = 7.805, *p* < 0.001), and benzaldehyde (*F*_(5,174)_ = 4.045, *p* = 0.002) (Figure 3D–H). Certain concentrations of limonene were particularly stimulatory while negative responses were observed at particular concentrations of isoamyl acetate and eugenol. Overall probing intensity was highest in benzaldehyde (mean = 22 ± 1.5 (SEM), n = 30 replicates/concentration) and limonene (mean = 21 ± 2.6, n = 15 replicates/concentration) and was lowest in vanillin (mean = 4.1 ± 0.06, n = 30 replicates/concentration) and anisole (mean = 4.9 ± 20.2, n = 45 replicates/concentration); the mean probing frequencies for the other test compounds fell between these extremes (menthol (mean = 14 ± 2.1, n = 30 replicates/concentration), isoamyl acetate (mean = 12 ± 1.3, n = 30 replicates/concentration) anisaldehyde (mean = 9 ± 1.0, n =30 replicates/concentration), and eugenol (mean = 9 ± 1.7, n = 15 replicates/concentration). For all compounds tested, there was no correlation between sex ratio and probing frequency (*F*_(1,153)_ = 0.17, *p* = 0.68, *r²* = 0.001).

### 3.2. Conditioned Response

**Figure 4:**
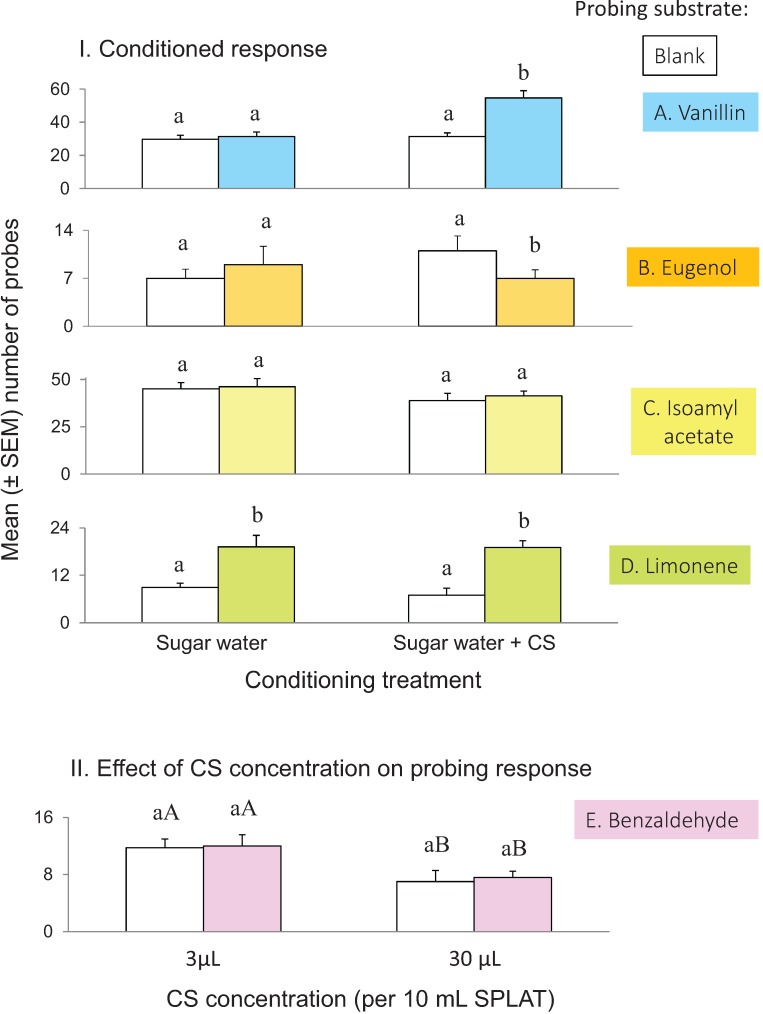
Results of conditioning experiments. In **I.**, different letters within each conditioning treatment pair are different at *p* ≤ 0.05. In **II.**, upper case letters indicate significance between concentration group pairs at* p* ≤ 0.05 (t-test). Sample sizes vary by test stimulus: N = 45, anisaldehyde; N = 40 for vanillin, isoamyl acetate, and anisole; and N = 30 for limonene and eugenol.

Psyllids experienced with vanillin probed the SPLAT + vanillin line significantly more than did the vanillin-naïve psyllids (*t* = 5.049, *p* < 0.001; α = 0.025) (Figure 4A). The probing levels of the vanillin-experienced psyllids to the blank SPLAT line was similar to that of the vanillin-naïve psyllids (*t* = 0.5049, *p* > 0.6; α = 0.025). Psyllids experienced on eugenol displayed significantly lower probing levels into the SPLAT + eugenol line than into blank SPLAT line (*t* = 2.329, *p* = 0.024; α = 0.05), however this negative response was not observed in eugenol-naïve psyllids (*t* = 1.1273, *p* = 0.25; α = 0.05) (Figure 4B, N = 30). There were no differences between the responses of naïve and experienced psyllids to isoamyl acetate (*t* = 0.3317, *p* > 0.74; α = 0.025) (Figure 4C), anisole (*t* = 0.9611, *p* = 0.34; α = 0.025), or anisaldehyde (*t* = 0.8740, *p* > 0.77; α = 0.025). Probing frequency was significantly higher on SPLAT + limonene than blank SPLAT for both limonene-naïve (*t* = 3.3374, *p* = 0.002; α = 0.05) and limonene-experienced psyllids (*t* = 4.815, *p* < 0.001; α = 0.05) (Figure 4D). For all test compounds tested, there was no correlation between sex ratio and probing frequency (*F*_(1,131)_ = 2.72, *p* =0.1; *r²* = 0.02).

### 3.3. Effect of Concentration on Conditioned Response

Psyllids could not be conditioned to benzaldehyde, either at a relatively lower (3 µL) or higher (30 µL) concentration (3 µL concentration: *t* = 0.0338, *p* = 0.79, α = 0.05; 30 µL concentration: *t* = 0.262, *p* = 0.97, α = 0.05) (Figure 4E). The response pattern observed in the learning test followed that of the innate response test in that the psyllids probed less frequently into the 30 µL concentration than into the 3 µL concentration (*t* = 3.634, *p* < 0.001, α = 0.05).

### 3.4. Effect of CS Mixing on Conditioned Response

**Figure 5:**
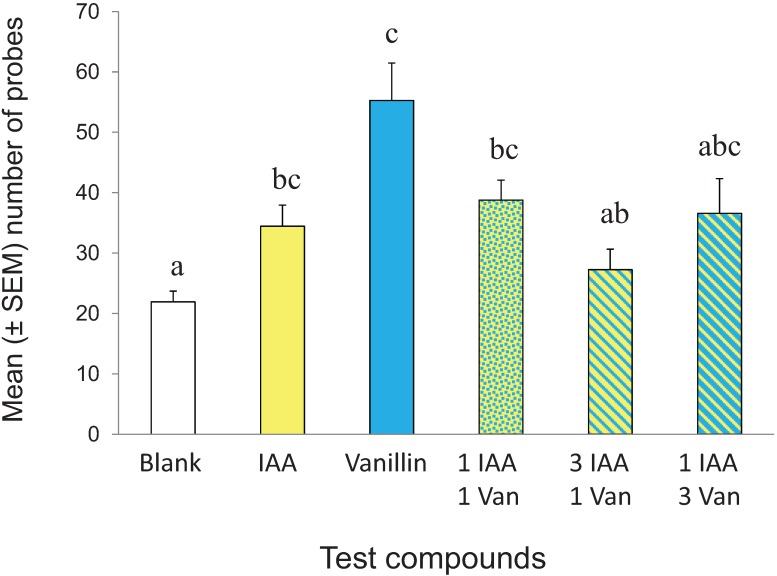
Response of vanillin-conditioned psyllids to vanillin (Van) isoamyl acetate (IAA), and binary mixtures of each in different proportions. N = 30 replicates for each treatment.

Following conditioning to vanillin, measurements were made of probing response to a blank SPLAT line and SPLAT lines with vanillin, isoamyl acetate and 1:1, 1:3, and 3:1 mixtures of vanillin and isoamyl acetate. Vanillin-experienced psyllids responded differently to the various test treatments (F_(5,173)_ = 6.451, *p* < 0.001) (Figure 5). Psyllids probed less frequently into the blank SPLAT line compared to lines with isoamyl acetate (*t* = 2.991, *p* < 0.005, α = 0.010), vanillin (*t* = 4.845, *p* < 0.001, α = 0.010), or the 1:1 isoamyl acetate-vanillin mixture (*t* = 4.220, *p* < 0.001, α = 0.010). The psyllids made numerically more probes into the SPLAT + vanillin line than into lines with either isoamyl acetate alone or the 1:1 isoamyl acetate-vanillin mix, though the differences were not significant (vanillin × isoamyl acetate: *t* = 2.790, *p* = 0.007, α = 0.005; vanillin × 1:1 mixture: *t* = 2.241, *p* = 0.029, α = 0.005). Significantly fewer probes were observed in the SPLAT line with the 3:1 isoamyl acetate-vanillin mixture than in the SPLAT line with vanillin alone (*t* = 3.778, *p* < 0.001, α = 0.005). Probing intensity was similar in the 1:1 and 1:3 mixes (*t* = 0.317, *p* = 0.73, α = 0.005). There were numerically fewer probes in the 3:1 isoamyl acetate-vanillin mix than in either the 1:1 and 1:3 mixes, though the differences were not significant (3:1 mixture × 1:1 mixture: *t* = 2.316, *p* = 0.024, α = 0.005; 3:1 mixture × 1:3 mixture: *t* = 1.332, *p* = 0.19, α = 0.005).

### 3.5. Visual Conditioning

**Figure 6:**
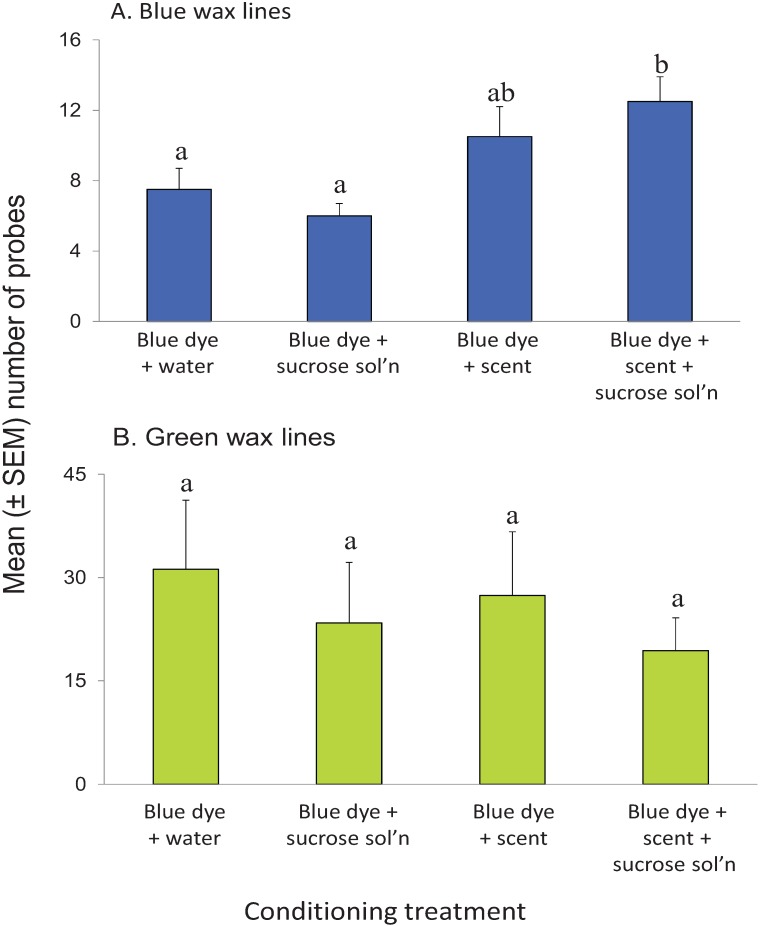
Probing responses of psyllids to blue (**A**) or green (**B**) SPLAT lines following experience on various combinations of visual, nutritive, and olfactory stimuli. Letters within the same row are different at *p* ≤ 0.05 (Planned comparisons with t-tests). Note differences in y-axis values between upper and lower graphs. N = 10 for each treatment.

Probing frequency on blue-colored SPLAT lines were measured following feeding experience on: (1) blue-colored water; (2) blue-colored sucrose solution; (3) blue colored water + citrus leaf scent; or (4) blue-colored sucrose solution + citrus leaf scent + sucrose. There were significant differences among treatments (*F*_(3,36)_ = 4.694, *p* = 0.0072) (Figure 6A). Higher probing frequencies were observed in psyllids that had fed on the scented blue-colored sucrose solution than in those that had fed on blue-colored water (*t* = 2.656, *p* = 0.024, α = 0.005) or blue-colored sucrose solution (*t* = 4.587, *p* = 0.001, α = 0.005). Probing responses of psyllids that had fed on scented blue water were numerically intermediate to those that had fed on blue-colored sucrose solution and scented blue sucrose solution. Psyllids from all of the conditioning treatments displayed vigorous probing responses into the control SPLAT lines that were colored bright green (Figure 6B). Overnight mortality increased significantly due to treatment (*F*_(3,36)_ = 11.984, *p* < 0.001). Only 18% of the psyllids survived overnight on the blue-colored water treatment, compared to 41% survival on scented blue-colored water. Psyllids provided with blue-colored sucrose solution or scented blue-colored sucrose solution had intermediate survivorship levels of, respectively, 25% and 33%.

## 4. Discussion

### 4.1. Innate Response

Limonene, benzaldehyde, and menthol elicited the highest levels of innate probing response while vanillin, anisol, and eugenol elicited the lowest responses, similar to conditions in which no test compound was presented. Probing frequencies were concentration dependent for limonene, menthol, benzaldehyde, eugenol, and isoamyl acetate. Since limonene occurs in the foliage of many *Citrus* species, a strong innate response to it could be anticipated. In our study area, limonene is a major component of the aroma of Mexican lime foliage, a minor component of the aroma of Meyer lemon foliage, and is absent from the aroma of orange jasmine foliage [15]. However, it is difficult to explain the high probing responses observed to benzaldehyde and menthol, neither of which occurs commonly in the foliage of the psyllid’s host-plants. In the case of menthol, perhaps *D. citri* response to monoterpenes is more generalized than would be expected. Further tests with groups of structurally similar and dissimilar monoterpenes would be needed to determine the specificity of *D. citri* response to monoterpenes as a whole. Benzaldehyde is a minor component of the floral fragrance of some *Citrus* species [43]. Since flowering in *Citrus* is often accompanied by leaf flush, especially in indeterminate species, low concentrations of benzaldehyde or other floral fragrance compounds, may signal leaf growth to the psyllid. Measuring psyllid response to floral volatiles of *Citrus* may reveal new stimulatory compounds. While low concentrations of eugenol appeared to suppress probing response, the highest dose elicited a response similar to the blank control. High concentrations of eugenol may have induced habituation or sensory adaptation. It may be beneficial to investigate which process is responsible.

### 4.2. Conditioning

There were no changes in behavior following conditioning to limonene. Elevated probing responses were displayed by both limonene-experienced and naïve psyllids. These results may suggest that *D.citri* possesses strong, innately biased responses to certain host-plant stimuli, such as limonene, that cannot be enhanced or otherwise modified by experience. Alternatively, the result could have been due to an experimental artifact because psyllids that fed on either Mexican lime or Meyer lemon would have experienced limonene while foraging prior to the experiment and were not truly limonene naïve. This would not have been the case for psyllids collected from orange jasmine. Further testing with psyllids reared only on orange jasmine, or other host-plants that do not produce limonene, would be required to verify this hypothesis.

Probing response to eugenol was depressed in the eugenol experienced-*versus* naïve psyllids. This result suggests that aversive conditioning may have occurred; *i.e.*, the psyllids had a negative reaction to eugenol during ingestion and learned to subsequently avoid it. While a previous study on *D. citri* response to essential oils in an olfactometer did not find eugenol to be repellent [44], the conflicting results may only reflect differences in test concentration and the discrete behaviors under investigation. Likewise, both benzaldehyde-naïve and experienced psyllids showed decreased probing levels to this compound at the higher test concentration. The psyllids did not display evidence of conditioning to isoamyl acetate at the concentrations tested.

Prior feeding on sucrose solution + vanillin strongly enhanced subsequent probing response to vanillin. This result along with that of the eugenol test, are significant because they demonstrate that *D. citri* has the cognitive capacity to learn to recognize chemosensory stimuli. In the case of vanillin, testing innate response did not indicate that it was stimulator to the psyllid; yet, vanillin had the strongest conditioned response. This result indicates that the olfactory capability of the psyllid may not be evident from simple dose-response tests that examine innately biased responses; conditioning tests may reveal hidden proclivities of the psyllid towards chemosensory compounds that intuitively would not be considered as stimulatory. In an applied sense, inclusion of these compounds to a scent lure mixture may enhance psyllid responsiveness to that mixture. As with menthol, the response to vanillin is somewhat inexplicable. One possibility is that vanillin is structurally similar to the primary monomers of lignin, aromatic alcohols called monolignols. Lignin is a constituent of the cell walls of fibers and other sclerenchymous cells in vascular bundles, the primary feeding site of *D. citri*. Vanillin’s molecular structure may be sufficiently similar to that of monolignols that it can function as a strong CS when coupled with an indicator of phloem, such as sucrose solution.

Another explanation is that if vanillin is not relevant in an ecological context to *D. citri*, then no innate biases exist that would preclude learning from occurring. This is consistent with the basic principles underlying classical conditioning—a conditioned response is produced from the repeated pairing of a biologically relevant unconditioned stimulus, like sucrose, with a neutral novel stimulus. All of the other compounds tested occur with some frequency either in host or non-host-plants in the psyllid’s environment and innate biases to them would be adaptive with respect to either finding or avoiding those plants that produce them. That *D. citri* has the capacity to associatively learn vanillin indicates that it is capable of some degree of cognitive plasticity with respect to host-plant selection. This capacity may be beneficial when it encounters new species of host-plants as a consequence of psyllid dispersal or the differential onset of host-plant growth within a given habitat. Alternatively, as has been observed in the potato psyllid, immature *D. citri* may learn to recognize certain cues produced by their natal host-plants and use these cues as adults in host-plant selection [35]. An interchange of innate and learned responses may be an effective means of enabling *D. citri* to locate suitable host-plants when they encounter novel habitats [45].

### 4.3. Mixing Test

The results of the CS mixing study suggest that an innate bias interfered with a conditioned response. Vanillin-experienced individuals were presented with mixtures of vanillin, a putatively neutral compound, and a fruit ester, isoamyl acetate. Vanillin-experienced psyllids responded similarly to isoamyl acetate alone and to the 1:1 and 1:3 isoamyl acetate-vanillin mixtures while their response to the 3:1 isoamyl acetate- vanillin mixture was similar to that of the blank control. Probing frequency was highest in response to vanillin alone, confirming the results of the initial conditioning experiment. The decreased response observed in the vanillin-experienced psyllids may have been a consequence of interference between the psyllid’s conditioned response to vanillin and their innate bias against isoamyl acetate. This type of interference may be external inhibition, or the phenomenon in which a learned response is suppressed in the presence of an aversive or distracting secondary stimulus. Regardless of the underlying cognitive mechanism, the results of the CS mixing experiment showed that, following conditioning, *D. citri* remained sensitive to the proportionality of chemosensory compounds presented to them.

### 4.4. Visual Learning

The results demonstrated that *D. citri* could learn not only chemosensory cues, but visual signals as well. The high mortality levels observed during conditioning indicate that the blue-colored solutions used in the feeding dishes were not stimulatory to the psyllids. In fact, it suggests that the color blue acted as a significant repellant. The addition of both scent and a nutritive stimulus, sucrose, was necessary to induce feeding and produce the association between the color blue and food. This result reinforces previous evidence for the synergistic effects of odor on psyllid behavior [16], in which psyllid response to gray visual targets was enhanced in the presence of foliar volatile compounds. It is known that *D. citri* displays color preferences [13]; that they can learn to associatively learn colors indicates that they possess a certain amount of behavioral plasticity towards visual stimuli. This, in turn, suggests that they can track variations and changes in leaf color. The ability to track visual cues could enhance their ability to select suitable (*i.e.*, flushing) host-plants in their local environment [45]. However, unlike with chemosensory stimuli, in which only a novel neutral stimulus was subject to modifications in perception, an innately repellant visual stimulus gained positive association relatively easily.

The results here and from other studies [14,15,16] indicate that host-plant selection by *D. citri* is driven primarily by innate responses to a combination of visual, olfactory, and gustatory stimuli and that presence of olfactory or gustatory cues can synergize psyllid response to visual stimuli. The circumstances under which conditioning to one or several different types of stimuli may enhance host-plant selection are not yet clear. Further studies of *D. citri* of different genders, life history stages and physiological conditions (*i.e.*; gravid, food-deprived) will be needed to determine under which conditions learning might be advantageous.

## 5. Conclusions

This study demonstrated that *D. citri* has the capacity to associatively learn to recognize both chemosensory and visual stimuli. The psyllids displayed both appetitive and aversive conditioning to two different chemosensory stimuli. They could also be conditioned to recognize a blue-colored probing substrate. The results confirmed those of an earlier study showing that the presence of olfactory cues enhanced psyllid response to neutral or weakly stimulating visual cues. The probing responses of psyllids conditioned on vanillin was diminished subsequently when the vanillin was mixed with various amounts of isoamyl acetate, showing that they are sensitive to the proportion of chemosensory components presented to them. They displayed strong to moderate innate biases to several of the test compounds; these were either monoterpenes emitted by foliage or aromatics or esters found in fruit. While innate responses are probably the psyllid’s primary behavioral mechanism for selecting host-plants, conditioning may enhance its ability to select host-plants during seasonal transitions and dispersal.
